# Mitogen-Activated Protein (MAP) Kinases in Plant Metal Stress: Regulation and Responses in Comparison to Other Biotic and Abiotic Stresses

**DOI:** 10.3390/ijms13067828

**Published:** 2012-06-21

**Authors:** Kelly Opdenakker, Tony Remans, Jaco Vangronsveld, Ann Cuypers

**Affiliations:** Centre for Environmental Sciences, Hasselt University, Agoralaan Building D, B-3590 Diepenbeek, Belgium; E-Mails: kelly.opdenakker@uhasselt.be (K.O.); tony.remans@uhasselt.be (T.R.); jaco.vangronsveld@uhasselt.be (J.V.)

**Keywords:** toxic metals, oxidative stress, reactive oxygen species (ROS), MAPK cascades, phosphatases, WRKY’s, ZAT12, antioxidative defense system, ethylene, jasmonic acid

## Abstract

Exposure of plants to toxic concentrations of metals leads to disruption of the cellular redox status followed by an accumulation of reactive oxygen species (ROS). ROS, like hydrogen peroxide, can act as signaling molecules in the cell and induce signaling via mitogen-activated protein kinase (MAPK) cascades. MAPK cascades are evolutionary conserved signal transduction modules, able to convert extracellular signals to appropriate cellular responses. In this review, our current understanding about MAPK signaling in plant metal stress is discussed. However, this knowledge is scarce compared to research into the role of MAPK signaling in the case of other abiotic and biotic stresses. ROS production is a common response induced by different stresses and undiscovered analogies may exist with metal stress. Therefore, further attention is given to MAPK signaling in other biotic and abiotic stresses and its interplay with other signaling pathways to create a framework in which the involvement of MAPK signaling in metal stress may be studied.

## 1. Introduction

Pollution of soils with toxic metals is a worldwide problem of great concern [1]. The high amount of metals, locally present in the environment, is mainly caused by mining and industrial activities [2,3]. However, the metal content of soils can also be increased due to the agricultural use of phosphate fertilizers, metal-containing pesticides and fungicides and sewage sludge, or irrigation with wastewater [4]. Metals like copper (Cu), iron (Fe), zinc (Zn), cobalt (Co) and nickel (Ni) are essential micronutrients required for normal plant growth and development [5,6]. Although they are essential, these elements can become toxic for plants when they exceed the concentrations normally present in the environment. Other elements, like cadmium (Cd), lead (Pb), and aluminium (Al), are nonessential and adversely affect biochemical reactions and physiological processes in plants [7]. Uptake and accumulation of these metals by food and feed crops brings these potentially toxic metals into the food chain for consumption by animals (cattle) and humans [4]. Several *in vitro* as well as *in vivo* epidemiological studies demonstrated that enhanced metal exposure poses a tremendous risk to human health [8–12].

In plants, the main visible symptoms of metal toxicity are leaf chlorosis and growth inhibition, caused by its interference with photosynthesis, mineral nutrition and the water balance [7,13,14]. At the cellular level, metals can disturb the cellular redox status by the production of reactive oxygen species (ROS) or interference with the antioxidant defense system [15–18]. Although ROS can react with biomolecules, which can get irreversibly damaged leading to necrosis and cell death, they can also act as signals in diverse biological processes in plants. In this way, they can influence signal transduction pathways and gene expression, suggesting that cells have evolved strategies to utilize ROS as signals for controlling various biological programs. ROS are suited to act as signaling molecules since they are small and can diffuse over short distances. Among the different ROS, only hydrogen peroxide (H_2_O_2_) can cross plant membranes and therefore directly function in cell-to-cell signaling [19,20]. In plant cells, H_2_O_2_ acts as a signal molecule involved in acclimatory signaling triggering tolerance to various abiotic and biotic stresses, like metal stress, ultraviolet radiation, salt stress, drought stress, light stress, temperature stress and in plant-pathogen interactions [21–26]. H_2_O_2_ has also been shown to act as a key regulator in a broad range of physiological processes, such as senescence, stomatal movement, programmed cell death, and growth and development [27–30]. Downstream signaling events associated with ROS sensing involve Ca^2+^ and Ca^2+^-binding proteins, such as calmodulin; the activation of G-proteins; the activation of phospholipid signaling, which results in the accumulation of phosphatidic acid; and/or activation of mitogen-activated protein kinase (MAPK) pathways [19,31]. H_2_O_2_ can modulate the activity and regulation of different components of MAPK cascades, such as protein phosphatases, protein kinases and transcription factors [32,33].

After a general introduction to MAPK cascades and their regulation, this review will specifically focus on MAPKinase signaling under metal stress conditions and the possible outcomes of this signaling cascades. Because H_2_O_2_ production is involved in multiple abiotic stresses, comparison with other known ROS-induced signaling pathways is discussed.

## 2. MAPKinases: Nomenclature and Classification

MAPK cascades are important signaling modules that convert signals generated from the receptors/sensors to cellular responses. They are composed of three protein kinase modules: MAPKK kinases (MAPKKK), MAPK kinases (MAPKK or MKK) and MAP kinases (MAPK or MPK). When MAPKKKs, serine/threonine kinases, are activated, they can phosphorylate MAPKKs via serine/threonine residues in the S/T-X_5_-S/T motif. MAPKKs are dual-specificity kinases that activate MAPKs through phosphorylation of both tyrosine and serine/threonine residues in the TXY motif. MAPKs are kinases that phosphorylate a variety of substrates, including transcription factors, transcription regulators, splicing factors and other protein kinases [34].

In *Arabidopsis*, 60 MAPKKKs, 10 MAPKKs and 20 MAPKs have been identified (Table 1). The MAPKKKs form the largest and most heterogeneous group of MAPK components. They can be divided in two large subgroups: the MEKK-type, for which MAPKKK function is provided, and the Raf-like kinases, for which MAPKKK function is not yet known. In *Arabidopsis*, examples of MEKK-like kinases are ANP1/2/3 and MEKK1. CTR1 and EDR1 belong to the Raf-like kinases [35].

MAPKKs (or MKKs) are the smallest group, only half as many as there are MAPKs. MAPKKs are probably able to activate multiple MAPKs, which suggests interplay between different signal transduction pathways occurring at this level. Plant MAPKKs are subdivided in four groups (A–D) based on sequence alignment. Group A MAPKKs seem to be involved in multiple abiotic stresses and cell division and are responsive to pathogens. MAPKKs belonging to group C are abiotic stress-responsive and function upstream of group A MAPKs, which play a role in environmental and hormonal responses [35].

*Arabidopsis* MAPKs (or MPKs) can be classified into two subtypes: those containing a TEY amino acid motif and those containing a TDY amino acid motif. The TEY subtype consists of three groups: A, B and C. MAPKs of group A are particularly involved in environmental and hormonal responses and examples of this group are MPK3 and MPK6. Group B MAPKs, to which MPK4 belongs, also play a role in environmental stress responses and seem to be involved in cell division. Little is known about group C MAPKs, but they appear to be circadian-rhythm-regulated. The TDY subtype forms group D. These MAPKs have a more extended C-terminal region in relation to groups A, B and C. On the other hand, they lack the C-terminal CD DOMAIN found in groups A, B and C, which serves as a docking site for MAPKKs, phosphatases and protein substrates. Group D MAPKs are shown to be induced by blast fungus and wounding [35].

The MAPKKKs are the most divergent group of kinases in the MAPK cascade. Therefore, different MAPKKKs can initiate similar MAPK cascades, which finally activate the same downstream MAPK. This is one of the mechanisms by which different stimuli converge onto one MAPK. The sharing of a single component by different cascades also leads to interaction between different pathways [35,36].

## 3. Regulation of MAPKinases

The outcome of a MAPK activation depends on the duration of its activation. The length of time that a MAPK remains active depends on the upstream specific regulation mechanisms, of which scaffolding (co-localization) and attenuation through phosphatases are best known. In addition, attention is given to lipid signaling which can initiate MAPK cascades.

### 3.1. Scaffolding

Specificity of different MAPK cascades functioning within the same cell is conferred by docking domains of scaffold proteins, which anchor different MAPK modules in one complex [37]. MAPK components themselves can also function as scaffolds. An example is the MAPKKK “Oxidative stress-activated MAP triple-kinase 1” (OMTK1), discovered in *Medicago sativa*. OMTK1 is able to activate MMK3 in response to H_2_O_2_ but not in case of cell treatment with yeast elicitor or 1-aminocyclopropane-1-carboxylic acid (ACC). Pull-down analysis between recombinant proteins showed that OMTK1 directly interacts with MKK3 and that both kinases are found together in a protein complex *in vivo*, suggesting that OMTK1 can determine specificity through its scaffolding function [38]. In *Arabidopsis*, no scaffold proteins of MAPK components with scaffold function are known to date.

### 3.2. Phosphatases

Negative regulation of MAPK cascades is performed by MAPK phosphatases, which dephosphorylate threonine and tyrosine residues within the activation motif of MAPKs. Three different forms of phosphatases were identified to date: protein tyrosine phosphatases (PTPs), protein serine-threonine phosphatases (PSTPs) or dual-specificity (Ser/Thr and Tyr) phosphatases (DSPs) [39]. In *A. thaliana*, members of all three classes are linked with MAPK inactivation, but dual-specificity MAPK phosphatases are the most important group because full inactivation of MAPKs requires dephosphorylation of both residues. The *Arabidopsis* genome encodes five possible candidate dual-specificity MAPK phosphatases (MKP1, MKP2, DsPTP1, PHS1 and IBR5) [39].

MKP1 was first identified by its involvement in genotoxic stress resistance. When 5-day-old seedlings were subjected to UV-C radiation (0.5–1 kJ/m^2^) or 50 mg L^−1^ methyl methane sulphonate, MKP1 was required for maintaining proper MAPK activity levels. Yeast-two-hybrid assays showed that MKP1 interacts with the stress-activated MAPKs MPK3, MPK4 and MPK6. Interaction of MKP1 with MPK6 was the most pronounced and MKP1 was reported to regulate MPK6 activity *in vivo* [40,41]. In addition, a role for MKP1 as a negative regulator of MPK3 and MPK6 activities was suggested in resistance against the bacterial pathogen *Pseudomonas syringae* and tolerance against UV-B and salt stresses [41–43]. Lee *et al.* [44] demonstrated that MKP1 activity increased after binding to calmodulin, indicating that Ca^2+^- and MAPK signaling are connected via the regulation of MKP1. MKPs can act together with other protein phosphatases to control MAPK activity. The protein tyrosine phosphatase 1 (PTP1), which was also shown to interact with MPK6, acts together with MKP1 to repress the biosynthesis of salicylic acid (SA) and camalexin, and pathogenesis-related gene expression, which makes plants more vulnerable to infection with *Pseudomonas syringae* [45].

Another MKP, MKP2, is also reported to bind and dephosphorylate MPK3 and MPK6 *in vitro* as well as *in vivo* [46,47]. After acute exposure to 500 ppb ozone, MKP2 acts as a positive regulator of the cellular redox status by repressing the activity of MPK3/6 [46]. In case of plant-pathogen interactions, MKP2 exerts differential and specific functions depending on the invading pathogen and is required for maintaining adequate levels of MPK3/6 activation. The appearance of bacterial wilting symptoms was delayed with one day in *mkp2* homozygous knockout plants infected with *Ralstonia solanacearum*. In contrast, *mkp2* knockout plants infected with *Botrytis cinerea* showed a systemic spread of the fungus throughout the whole plant after 15 days of inoculation, whereas in wild type plants lesions were local and restricted to the inoculated leaves [47].

### 3.3. Lipid Signaling

Besides the regulation of MAPKs by protein phosphatases, MAPK cascades can also be regulated via lipid signaling. Plasma membrane-associated phospholipase D (PLD) enzymes release phosphatidic acid (PA) from phosphatidylcholine, phosphatidylethanolamine and phosphatidylglycerol. PA has been implicated as a secondary messenger in many different stress responses, such as the production of ROS [48].

In *Arabidopsis*, Yu *et al.* [49] showed that PLD-derived PA binds to MPK6, leading to its activation during salt stress. Activated MPK6 is responsible for the phosphorylation of the Na^+^/H^+^ antiporter SOS1, which reduces Na^+^ accumulation in *Arabidopsis* leaves. In *Arabidopsis*, Anthony *et al.* [50] reported that binding of PA to “3-phosphinositide-dependent protein kinase 1” (PDK1) stimulates phosphorylation and activation of the serine/threonine protein kinase “Oxidative-signal inducible 1” (OXI1). Activity of OXI1 was induced within 30 min after treatment of *Arabidopsis* plants with plant growth factors, like auxin and cytokinin, suggesting a role for OXI1 in plant growth and cell division. Rentel *et al.* [51] revealed that OXI1 is involved in H_2_O_2_-dependent activation of MPK3/6 in ROS-dependent processes such as root-hair elongation and basal resistance to the fungal pathogen *Peronospora parasitica*. In addition, PA-stimulated activation of the PDK1/OXI1/MPK6 pathway was shown to promote plant growth in *Arabidopsis* seedlings after co-cultivation with the endophytic fungus *Piriformospora indica* [52]. Activation of MAPKs by OXI1 is mediated by serine/threonine protein kinases of the Pto-interacting 1 (PTI1) like family or NDP kinase 2 (NDPK2) [53–55]. However, further research is required to unravel the OXI1–MAPK cascade.

## 4. Role of MAPK Cascades in Stress Response Signaling

MAPK cascades are involved in normal cell metabolism like physiological, developmental and hormonal responses [34,56,57]. However multiple studies have shown that MAPK cascades play important roles in plant responses to biotic and abiotic stresses, such as pathogen infection, wounding, low temperature, drought, hyper- and hypo-osmolarity, high salininty, mechanical stress, metals and ROS [57–59].

### 4.1. MAPK Cascades Are Involved in Metal Stress

Several authors reported the involvement of MAPK signaling in metal stress for different plant species (Table 2). In general, mRNA as well as activity levels are increased quickly after metal exposure, ranging from 5 min to 1 h, and activation of MAPKs is transient. In *Arabidopsis*, it is proven that MPK3 and MPK6 are activated in response to short-term exposure (less than 1 h) to CdCl_2_ concentrations as low as 1 μM, via the accumulation of ROS [60]. However, so far, evidence for the involvement of a complete MAPK cascade pathway in metal stress responses is rather scarce in plants.

Rao *et al.* [61] predicted a possible MAPK cascade in rice namely OsMKK4/OSMPK3. Two weeks old rice plants exposed to 50 μM arsenite showed increased transcript levels of *OsMPK3* in leaves and roots already after 30 min of exposure. These results were confirmed at the protein level: activity of OsMPK3 was elevated within 3h. Gene expression levels of *OsMKK4* were also elevated in leaves and roots after 3 h exposure to arsenite. In *Medicago sativa* roots, transient activation of MAPKs (SIMK, SAMK, MKK2 and MMK3) was rapidly induced (less than 10 min) after exposure to 100 μM CuCl_2_, whereas treatment with 100 μM CdCl_2_ delayed this profile. In addition, transient expression assays in *Arabidopsis* protoplasts with HA-tagged SIMK, SAMK, MMK2 and MKK3, and a myc-tagged MAPKK (SIMKK), showed that SIMKK specifically activated SIMK and SAMK after exposure to 100 μM CuCl_2_ [62]. Opdenakker *et al.* [21] showed that 24 h exposure of *Arabidopsis thaliana* seedlings to environmental realistic concentrations of Cu and Cd increased transcript levels of MAPKinases in a time-dependent manner. After 2 h of exposure to 2 μM Cu, gene expression of *OXI1*, the MAPKKK “Arabidopsis NPK1-like protein kinase 1” (*ANP1*) and the MAPKs *MPK3* and *MPK6* was already affected in the roots and leaves of *Arabidopsis* plants. After exposure to 5 μM Cd, no changes in gene expression of these enzymes were observed before 24 h. These changes in gene expression seemed to be related to the production of H_2_O_2_ by these metals, either directly and fast by Cu (Fenton-HaberWeiss reactions) or indirectly and delayed by Cd (e.g., via NADPH oxidases).

Activation of ANP1 and OXI1 by H_2_O_2_ and induction of a phosphorylation cascade involving MPK3 and MPK6 has been reported before in *Arabidopsis* leaf cells and whole plants [51,63]. Application of 200 μM H_2_O_2_ to *Arabidopsis* protoplasts, increased the activity of ANP1, MPK3 and MPK6 within 10 min. Co-transfection of protoplasts with ANP1 and MPK3 or MPK6 revealed that ANP1 could further enhance the activity of MPK3 and MPK6 after H_2_O_2_ treatment [63]. Rentel *et al.* [51] showed that gene expression of *OXI1* was already enhanced after 30 min in 7-day-old seedlings treated with 10 mM H_2_O_2_ and *oxi1* knockout mutants failed to activate MPK3 and MPK6 after treatment with H_2_O_2_. Additionally, a toxicity test based on primary root elongation showed that *oxi1* and *mpk6* knock-outs were more tolerant to excess Cu, but not Cd, suggesting that OXI1 and MPK6 play important roles in the observed stress response following Cu exposure [64].

However, knowledge about the downstream signaling targets of MAPKs is rather scarce under metal stress. Roelofs *et al.* [65] compared known signaling pathways induced by metals stress as well as by other abiotic stresses (cold, heat, salt, drought) between soil invertebrates and plants. They showed that all abiotic stresses switched on more than one stress-responsive pathway, seen in the overlap of transcription factors used by each stressor, and they speculated that bZIP, MYB and MYC transcription factors could be downstream targets of MAPK signaling in plant metal stress.

**Figure 1:**
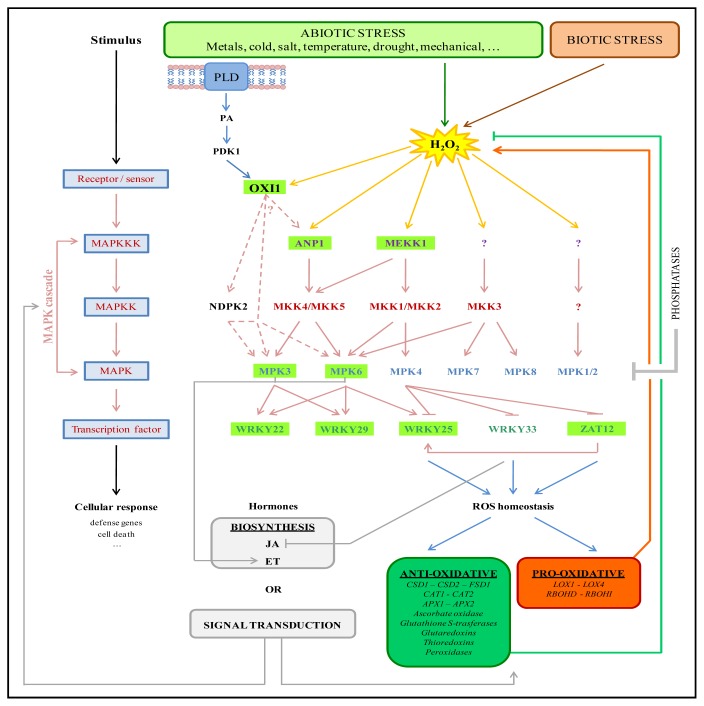
Overview of the different MAPK cascades known to function in stress responses as well as their regulation and possible downstream outcomes (signaling components involved in metal stress are highlighted in green). Production of ROS is a common phenomenon between different biotic and abiotic stresses. In particular H_2_O_2_ can act as an intra- and intercellular signaling molecule activating MAPK cascades. Via lipid signaling or phosphorylation of OXI1, ROS production can be linked to MAPK activation. MAPK signaling pathways induce gene expression of stress-responsive genes through the activation of transcription factors. On one hand, MAPK cascades influence the cellular redox status by activating gene expression of antioxidative or pro-oxidative enzymes. In this way, MAPK signaling can attenuate or amplify the initial ROS signal. On the other hand, MAPKs interfere with hormone signaling and biosynthesis leading to activation of downstream stress responses. Inactivation of MAPK pathways occurs through phosphatases, which dephosphorylate MAPKs, resulting in the disruption of the MAPK signal and are therefore important in the regulation of downstream responses.

Interplay between the MAPK pathways activated by metal stress and the ones used by other stresses probably exists because ROS generation, which is known to induce MAPK signaling, is common to other abiotic and biotic stress responses (Figure 1).

### 4.2. Comparison to MAPK Pathways Involved in Other Stress Responses

In *Arabidopsis* protoplasts, Kovtun *et al.* [63] showed that the MAPKKK ANP1, induced by H_2_O_2_, activated the downstream MAPKs MPK3/6. The MAPKKs involved in the activation of MPK3/6 by ANP1 could be MKK4 and MKK5. Ren *et al.* [79] reported that transgenic *Arabidopsis* plants, expressing *MKK4* and *MKK5* under the control of a steroid-inducible promoter, were able to activate MPK3/6, resulting in cell death. The protein kinase OXI1 is, as already mentioned above, involved in H_2_O_2_-dependent activation of MPK3/6 [51]. Moreover, *oxi1* knockout mutants showed defects in ROS-dependent developmental processes such as root-hair elongation, and in ROS-dependent basal resistance to the fungal pathogen *Peronospora parasitica*. In what way OXI1 activates MPK3 and MPK6 remains to be addressed, although it is suggested that this activation may be modulated by NDP kinase 2 (NDPK2) [55,59]. Exposure of 2-week-old *Arabidopsis* plants to 4 mM H_2_O_2_ strongly increased gene expression of *NDPK2* within 30 min and up to 12h, suggesting that NDPK2 functions in ROS homeostasis. Furthermore, overexpression of *NDPK2* resulted in lower levels of ROS as compared to wild type plants and conferred enhanced tolerance to environmental stresses that induce ROS generation, such as freezing during 1h or exposure to 50 mM NaCl for 3 weeks. Specific interaction between NDPK2 and MPK3/6 was discovered using yeast two-hybrid and *in vitro* protein pull-down assays. NDPK2 was also shown to enhance the myelin basic protein phosphorylation activity of MPK3 *in vitro* [55].

Nakagami *et al.* [80] reported on another *Arabidopsis* MAPKKK, MEKK1, which is also regulated by H_2_O_2_ and was found to activate the MAPK MPK4 in response to treatment of *Arabidopsis* protoplasts with 2 mM H_2_O_2_ during 5 min. Ten-day-old *mekk1* knockout plants showed a deregulated expression of genes involved in cellular redox control, like glutathione S-transferases, NADPH oxidases and ascorbate peroxidases, and accumulated ROS, suggesting that MEKK1 is necessary for normal redox homeostasis of the cell. The MAPK cascade MEKK1-MKK1/MKK2-MPK4 was earlier identified using yeast two-hybrid and complementation analysis of yeast mutants [81,82]. Later, it was shown that MKK1 phosphorylates MPK4 *in vitro* as well as *in vivo* and that this cascade is operating in different environmental stresses such as low temperature, low humidity, hyper-osmolarity and mechanical stress [83–85]. A microarray study with 14-day-old *mekk1*, *mkk1/2* and *mpk4* knockout *Arabidopsis* plants performed by Pitzschke *et al.* [86] showed that the MEKK1-MKK1/2-MPK4 cascade is a key regulator of ROS- and SA-initiated stress signaling. However, they also suggest that MEKK1 can activate another pathway, independent of MKK1/2 and MPK4, and that MKK1/2 is not only regulating MPK4, but most likely also other MAPKs like MPK3/6. Asai *et al.* [87] showed that in protoplasts treated with 100 mM of a bacterial flagellin peptide (flg22), MEKK1 activated MPK3/6 through MKK4/MKK5, leading to the expression of early-defense response genes. Activation of the MEKK1-signaling cascade by flg22 is mediated by receptor-like kinases (RLKs), which are also reported to be regulated by Cd stress [88]. Recently, MKK4 was also identified to be involved in abiotic salt stress responses, as a regulator of MPK3 activity [89]. In contrast, studies performed with *mekk1* knockout plants instead of the protoplast system used by Asai *et al.* [87] reported that 14-day-old *mekk1* knockout plants did activate MPK3 and MPK6 activity within 10 min after treatment with flg22, but failed to induce MPK4 activity. These results indicate that probably more alternative pathways exist to activate MPK3/6 in the absence of MEKK1 [90]. MKK2, activated by MEKK1, was observed to directly target MPK4 and MPK6 in cold and salt stress [91]. Plants overexpressing *MKK2* exhibited constitutive MPK4 and MPK6 activity, showing increased freezing and salt tolerance while *mkk2* knockout plants were impaired in MPK4 and MPK6 activation, showing hypersensitivity to salt and cold stress. These studies demonstrate that MEKK1 can integrate different stress signals and ensures stress-specific responses by activating different downstream MAPKs.

Recently, another MAPK component, able to integrate different stress signals, was identified. MKK3 acts as upstream activator of MPK7, which induces target genes such as *PR1* in the defense response against *Pseudomonas syringae.* In *Arabidopsis* protoplasts, transiently expressing *MKK3* and *MPK7*, MKK3-mediated activity of MPK7 was only induced after application of 4 mM H_2_O_2_ during 5 or 15 min, whereas treatment with 1 μM flg22 had no effect on the MPK7 activity. These data led to the observation that the MKK3-MPK7 cascade is induced by H_2_O_2_-mediated inhibition of the proteasome-dependent degradation of MKK3 [92]. In contrast, the MKK3-MPK6 pathway functions in jasmonic acid (JA) signaling. In 2-week-old wild type plants, MPK6 activity was enhanced directly after treatment with 50 μM JA. This activity was reduced in *mkk3* knockout plants and higher in *MKK3* overexpressing plants exposed to JA. In addition, JA-dependent inhibition of root growth and induction of *PDF1.2* and *VSP2* expression was regulated by MKK3-MPK6 [93]. In the case of mechanical stress, MKK3 is, together with Ca/CaM, responsible for the full activation of MPK8, which negatively regulates the expression of *RBOHD* (NADPH-oxidase). RBOHD plays an important role in ROS generation and the ROS signal provided by RBOHD is involved in the induction of wound-inducible marker genes, such as *OXI1* and *ZAT12*. Therefore, the negative regulation of *RBOHD* by MPK8 is required for appropriate production of ROS during mechanical stress responses [94]. In addition, Ortiz-Masia *et al.* [95] showed a rapid (within 15 min) activation in 4-week-old *Arabidopsis* plants upon treatment with 5 mM H_2_O_2_ as well as by other stress signals such as mechanical stress or application of 50 μM JA or 100 μM abscisic acid (ABA).

## 5. MAPK Cascades Regulate Stress Responses by Activation of Gene Transcription

### 5.1. Transcription Factors

MAPK cascades have the possibility to regulate gene transcription by activation or repression of transcription factors. Popescu *et al.* [96] used high-density *Arabidopsis* protein microarrays to identify *in vitro* novel MPK targets. They observed that the largest group of possible MPK targets identified in their screen represent transcription factors. Coexpression of WRKY and TGA transcription factors with specific MKK/MPK modules showed that these transcription factors are also phosphorylated *in vivo*.

WRKY proteins bind to W-box DNA elements (containing a TGAC core sequence) and act both as positive and negative regulators of target gene expression. WRKY family members are subdivided into three groups based on the number of WRKY domains and certain features of the zinc finger-like motifs (for a review, see [97] and references therein). WRKY transcription factors are known to be involved in diverse biotic and abiotic stresses (Figure 1). Short-term exposure of 3-week-old *Arabidopsis* plants to 2 μM Cu, induced gene expression of *WRKY22*, *WRKY25* and *WRKY29* already after 2h of exposure in leaves and roots. In contrast, after 24 h of exposure to 5 μM Cd only the gene expression of *WRKY25* and *WRKY29* was affected. These data suggest that these transcription factors play an essential role in regulation of the stress response upon metal exposure [21]. Involvement of these WRKY transcription factors was also reported in other stress conditions confirming the hypothesis that use of these transcription factors is not specific for metal stress signaling but is common between different biotic and abiotic stress responses. Investigation of transcriptome data generated from ROS-related microarray experiments, showed that induction of WRKY22 gene expression is also related to singlet oxygen, ozone and superoxide production [98]. More recently, *WRKY22* gene expression was reported to be induced by H_2_O_2_ in leaf senescence [99]. Transcript levels of *WRKY22* were induced 1h after application of 3% H_2_O_2_ or by dark treatment already after 1 day. The involvement of WRKY22 in dark-induced leaf senescence was investigated by comparing *wrky22* knockout plants and *WRKY22* overexpressing plants with wild type plants in relation to cell death, chlorophyll content and expression of senescence-associated genes [99]. The flagellin induced MAPK cascade MEKK1-MKK4/MKK5-MPK3/MPK6 is known to activate WRKY22 and his close homolog WRKY29. They positively regulate gene expression of disease resistance genes to confer resistance to both bacterial and fungal pathogens [87].

*In vitro* and *in vivo* interaction studies revealed a MPK4 substrate MKS1 (MAP kinase 4 substrate 1), which functions in coupling MPK4 to WRKY25 and WRKY33. In this way, WRKY25/33 function in the regulation of pathogen defense responses by repression of SA-dependent resistance. Indeed, *Arabidopsis* plants overexpressing *WRKY25* showed increased disease symptoms together with an invasive bacterial growth after inoculation with *Pseudomonas syringae* during 3 and 4 days. This was due to reduced expression of *PR1*, a molecular marker for SA-mediated defense signaling [100,101]. In addition, studies pointed out a role for WRKY25 in the modulation of gene transcription during heat and salt stress [102,103]. Transcript levels of *WRKY25* were induced within 30 min in *Arabidopsis* plants exposed to 42 °C. Five-day-old *wrky25* knockout plants showed an increased inhibition of root elongation when exposed to 45 °C for 4 h, whereas 3-week-old *wrky25* knockout displayed greater electrolyte leakage after 4h exposure to 42 °C, indicating the positive role of *WRKY25* in thermotolerance [102]. Treatment of 3-week-old *Arabidopsis* plants with 150 mM NaCl during 6 and 24 h induced gene expression of *WRKY25* as well as *WRKY33* strongly. Root growth was stimulated in *WRKY25* and *WRKY33* overexpressing plants in the presence of 100 mM NaCl, suggesting the involvement of these transcription factors in tolerance against salt stress [103].

The zinc finger transcription factor (C2H2-type) *ZAT12* is also involved during metal stress responses. As was also observed for the WRKY transcription factors, transcript levels of *ZAT12* were elevated after 2 h in roots of 3-week-old *Arabidopsis* plants exposed to 2 μM Cu, whereas *ZAT12* expression in 5 μM Cd-exposed roots was not increased before 24 h. In leaves, gene expression of *ZAT12* was increased after 24h in both Cu- and Cd-exposed plants [21]. Besides its involvement in metal stress, ZAT12 was also found to respond at the transcriptional level to other abiotic and biotic stresses. A comparison of microarray profiles of 6-week-old wild type and catalase-deficient (20% residual catalase activity) *Arabidopsis* plants exposed to high light for at least 3 h, identified ZAT12 as a H_2_O_2_-responsive transcription factor [24,104]. This observation was confirmed by the results of a microarray study performed on *Arabidopsis* cell cultures exposed to 20 mM H_2_O_2_ during 1.5 and 3 h [105]. Mechanically stressed leaves of 4-week-old *Arabidopsis* seedlings showed significantly increased transcript levels of *ZAT12* after 30 min. This increase in gene expression was still visible after 6h of stress [106]. Activation of *ZAT12* transcription was also reported to occur in 3-week-old *Arabidopsis* plants in response to cold (4 °C, 2 h), heat (38 °C, 1 h), salt (150 mM NaCl, 4 h) and drought (75% relative water content) [107].

### 5.2. Regulation of the Cellular Redox Status by MAPK Cascades

Metal stress, as well as other biotic and abiotic stresses, is known to disrupt the cellular redox status by stimulating the production of ROS or affecting the antioxidative defense system of the cell. Signaling via MAPK cascades can influence both sides of the redox balance (Figure 1).

Pitzschke *et al.* [86] showed that the MEKK1-MKK1/MMK2-MPK4 pathway negatively controlled the gene expression of *WRKY25*, *WRKY33* and *ZAT12*. Expression of oxidative stress responsive genes like the Cu/Zn superoxide dismutase *CSD1*, the catalase *CAT2*, the NADPH oxidase *RBOHI*, and certain glutaredoxins and thioredoxins, was also altered in *mpk4* knockout plants. This suggests that the MEKK1-MMK1/MMK2-MPK4 pathway regulates ROS homeostasis via the transcription factors WRKY25/33 and ZAT12. Additionally, in the cases of heat or salt stress, WRKY25 and WRKY33 were reported to influence the gene expression of ROS responsive genes [102,103]. Exposure of 21-day-old *wrky25* knockout plants to 42 °C for 30, 60 and 120 min showed lower transcript levels of the ascorbate peroxidases *APX1* and *2* as compared to wild type plants. These data indicate that WRKY25 can positively regulate the expression of two oxidative stress-responsive genes *APX1* and *APX2* [102]. Microarray studies on salt-exposed (150 mM, 6 h) *wrky33* knockout plants revealed glutathione-S-transferases, class III peroxidases and the lipoxygenase *LOX1* as possible targets of WRKY33 transcriptional activity [103]. Because WRKY25 and WRKY33 share very similar protein structures [97], it is possible that they are involved in the regulation of the same genes. For example, Li *et al.* [108] showed that WRKY25 and WRKY33, together with WRKY26, work coordinately to induce thermotolerance in plants.

Studies with *ZAT12* overexpressing as well as knockout plants revealed that ZAT12 is responsible for the induction of oxidative stress-related transcripts, like *APX1*, *CSD1*, *CSD2*, *RBOHD*, *LOX4* and several glutathione S-transferases, while reducing transcript levels of the iron superoxide dismutase *FSD1*, an L-ascorbate oxidase, several peroxidases and glutaredoxins [107,109–111]. These data suggest that ZAT12 is important in facilitating plants to cope with oxidative stress. ZAT12 was also identified as inducer of WRKY25 during oxidative stress, meaning that WRKY25 acts downstream of ZAT12 to control its target genes [102,109]. Nakagami *et al.* [80] suggested a role for the MEKK1-MPK4 pathway in the negative regulation of ZAT12 under oxidative stress conditions. Exposure of *mekk1* and *mpk4* knockout plants to 10 mM H_2_O_2_ during 1h resulted in increased *ZAT12* transcript levels as compared to wild type plants. Gene expression of *CAT1*, not *CAT2* nor *CAT3*, is regulated by MKK1-mediated H_2_O_2_ production during different types of abiotic stress, such as drought and salt stress, and is related to ABA-signaling [112]. In addition, transcript levels of *CAT1*, not *CAT2* nor *CAT3*, were reduced in 2-week-old *mkk1* knockout plants and increased in *MKK1* overexpressing plants as compared to wild type plants after 4h exposure to 300 mM NaCl, drought stress (dehydration of plants to 80% of their original fresh weight followed by incubation at 100% relative humidity at 25 °C) or 0.1 mM ABA. Furthermore, production of H_2_O_2_ was abolished in *mkk1* knockout plants and higher in *MKK1* overexpressors treated with 300 mM NaCl, drought or 100 μM ABA. MKK1 mediates ABA-induced *CAT1* expression via MPK6 [113]. As shown for *mkk1* knockout and *MKK1* overexpressing plants, *CAT1* transcript levels were reduced in 2-week-old *mpk6* knockout plants and elevated in *MPK6* overexpressors exposed to 100 μM ABA. ABA treatment also inhibited H_2_O_2_ accumulation in *mpk6* knockout plants.

### 5.3. MAPK Cascades Interfere with Hormone Signaling

Besides their role in physiological processes, ethylene and JA were originally identified as stress hormones essential for plant defense against a variety of abiotic and biotic stresses, such as ozone, UV radiation, mechanical stress, chemicals, metals, drought, extreme temperatures, insect and pathogen infection [114,115]. In the past years, more and more evidence was presented to indicate that MAPK cascades can play a role in ethylene and JA signaling (Figure 1).

#### 5.3.1. Ethylene

MAPkinases have been reported to be involved in ethylene biosynthesis as well as in ethylene signaling. Several data indicate that MPK6 is involved in ethylene biosynthesis. It was reported in twelve-day-old *Arabidopsis* plants that MPK6, induced by flg22 or mechanical stress, is responsible for the phosphorylation of two ACC synthases, ACS2 and ACS6. Conversion of S-adenosyl-L-methionine to ACC by ACC synthases is the rate-limiting and major regulatory step in stress-induced ethylene production. Phosphorylation of ACS2 and ACS6 stabilizes the ACS proteins, leading to an elevated ACS activity and consequently an elevated ACC and ethylene production [116]. In addition, the MKK9-MPK3/6 pathway was shown to function in ethylene biosynthesis. Constitutive expression of MKK9 induced accumulation of ethylene through activation of MPK3/6 and consecutive positive regulation of *ACS2* and *ACS6* gene expression [117].

In contrast, other studies report that MAPKinases are involved in ethylene signal transduction rather than in ethylene biosynthesis. The function of the different components in the ethylene signaling pathway was recently reviewed by Shan *et al.* [118] and will be discussed here briefly. Binding of ethylene to its receptor, ETR1 (Ethylene Response 1), releases CTR1 (Constitutive Triple Response 1), which in the absence of ethylene leads to degradation of EIN2 (Ethylene Insensitive 2), inhibiting downstream signal transduction. Therefore, binding of ethylene leads to accumulation of EIN2 activating a transcriptional cascade, initiated by the transcription factor EIN3 (Ethylene Insensitive 3), regulating transcription of ethylene responsive genes [118]. The first evidence for the involvement of a MAPK cascade in plant ethylene signaling came with the discovery of the first gene in the ethylene signal transduction pathway, *CTR1*, a negative regulator of ethylene responses. Indeed, the predicted protein sequence of CTR1 showed similarity to the mammalian Raf kinase, a MAPKKK [119]. More recently, Novikova *et al.* [120] showed that a protein with similarities to a MAPK was activated by exogenous treatment of 6-week-old *Arabidopsis* plants with 1 μL L^−1^ ethylene during 1h. Treatment (10 min) of *Arabidopsis* wild type plants with 1mM ACC, the immediate precursor of ethylene in the biosynthesis pathway, identified the activation of MPK6. Mutant screenings showed that this activation of MPK6 by ACC is mediated by ETR1 and CTR1, but not by EIN2 or EIN3. These results place MPK6, as a positive regulator of ethylene responses, downstream of CTR1 and upstream of EIN2 in the ethylene signal transduction pathway [121]. A study by Yoo *et al.* [122] reported that the MAPK cascade MKK9-MPK3/6 functions downstream of CTR1, is activated upon binding of ethylene to ETR1 and is able to phosphorylate and stabilize EIN3 leading to transcription of ethylene responsive genes. Yeast-2-hybrid and fluorescence resonance energy transfer identified the *in vitro* as well *in vivo* interaction of MPK6 with an ethylene response factor ERF104. The MPK6/ERF104 complex is disrupted by flg22-induced ethylene production, leading to phosphorylation and activation of ERF104. Microarray analysis of *ERF104* overexpressing plants identified the ERF104 stimulated targets as pathogenesis related (*PDF1.2*) or involved in further signal amplification of defense signaling (*MKK4*, *RBOHD*, *WRKY33*) [123].

#### 5.3.2. Jasmonate

Similar to ethylene, MAPKs are indicated to play a role in the biosynthesis of JA as well as in JA signaling. In case of salt stress, transcripts of two genes involved in JA biosynthesis, *OPR1* and *OPR2*, were shown to be more abundant in *wrky33* knockout plants, suggesting that MAPK cascades involving WRKY33 can downregulate JA biosynthesis during heat stress [103]. Earlier, we mentioned that activation of transcription factors WRKY33 or ZAT12 by MAPK cascades induced gene expression of lipoxygenases, *LOX1* and *LOX4* respectively. Involvement of lipoxygenases in metal stress was also reported. Transcript levels of *LOX2* were significantly induced in leaves of *Arabidopsis* plants after short term exposure to 2 μM Cu or 5 μM Cd. In the roots, a metal-specific upregulation of *LOX3* and *LOX4* was observed after Cu exposure, whereas transcript levels of *LOX5* were specifically downregulated. In contrast, *LOX1* and *LOX6* gene expression was responsive to both Cd and Cu [21,124]. Further, expression of *LOX1* and *LOX2* is compromised in roots and leaves of non-exposed *mpk6* knockout plants [125]. Lipoxygenases catalyze the first step in JA biosynthesis, namely the oxygenation of α-linolenic acid to hydroperoxides [115]. Earlier, LOX2 was identified as a lipoxygenase responsible for initiating JA biosynthesis upon wounding [126]. Taken together, these data suggest that MAPK cascades can initiate JA biosynthesis via transcriptional control of LOXes.

On the other hand, studies reported that MAPKs would function in JA signal transduction. Leon *et al.* [127] stated that reversible protein phosphorylation is involved in JA signaling. JA-dependent induction of wound-inducible genes was stimulated by treatment of *Arabidopsis* plants with the protein kinase inhibitor staurosporin while treatment with the protein phosphatase inhibitor okadaic acid repressed this gene expression. These results suggest a negative regulation of the JA downstream pathway by protein kinase cascades. Petersen *et al.* [128] showed that *mpk4* knockout plants are impaired in expression of *PDF1.2* and *THI1.2*, two JA-responsive genes. Moreover, treatment of 4-week-old *mpk4* knockout plants with 50 μM methyl-JA for 48 h failed to induce *PDF1.2* or *THI1.2* transcript levels, indicating that MPK4 is involved in JA signaling. In addition, the MKK3-MPK6 pathway functions in JA signaling in *Arabidopsis* as a negative regulator of the downstream transcription factor MYC2. Treatment of *mkk3* and *mpk6* knockout as well as *MKK3* and *MPK6* overexpressing plants with 50 μM JA during 12 h, showed that the MKK3/MPK6 pathway induced or reduced the transcript levels of *MYC2* respectively [93].

## 6. Conclusions

MAPK signaling plays a central role in plant metal stress responses. MAPkinases are activated by ROS production, induced upon metal stress, and convert the perception of metals to intracellular signals to the nucleus, where appropriate responses are initiated. However, MAPK cascades are not specific for a single stress condition. One MAPK cascade can be used by different biotic and abiotic stresses and interplay between different pathways is possible. In metal stress, the function of MAPK cascades is poorly understood while knowledge about metal signaling and more specific their downstream targets is essential for understanding plant responses to metal stress. Therefore, in future research it is important to focus on the functional analysis of MAPkinases in plant metal stress. For this purpose, mutants (knock-outs, overexpressors) of the different MAPKKKs, MAPKKs and MAPKs should be investigated under metal exposure in a single and multipollution context. Interaction between the different MAPKinase modules and the possible transcription factors activated by MAPKs can be identified by the use of functional protein microarrays [96] or phosphoproteomics [129]. Specific genes targeted by these transcription factors in their turn can be resolved using different molecular strategies. Better insight into plant metal stress responses and their regulation opens future perspectives to investigate the complexity of signaling modules in plant responses facing a globally changing environment.

## Figures and Tables

**Table 1:** Classification of mitogen-activated protein (MAP) Kinases. In the first and second column, the different MAPKinase modules and the amino acid sequences phosphorylated by them are presented. Then MAPKKKs, MAPKKs and MAPKs are classified according to their phosphorylated amino acid sequence and placed in groups A to D based on sequence alignment. In the final two columns, examples of MAPKinases belonging to each group as well as the plant responses, in which they are involved, are provided. MAPkinases indicated in bold are discussed in this review.

Classification of MAPKinases					

MAPKinase	Amino acids phosphorylated by the kinase	Phosphorylated amino acid motif	Group	Examples	Response to/in
MAPKKK	Serine/threonine	MEKK1-type	A	**MEKK1**, MEKK2, MEKK3, MEKK4	Redox control–oxidative stress; Abiotic stresses: drought, salt, mechanical; Biotic stress: bacterial pathogens; Hormones: salicylic acid.
				**ANP1**, ANP2, ANP3	Redox control–oxidative stress; Biotic stress: bacterial pathogens; Hormones: auxin; Cytokinesis.
		Raf-like	B	EDR1, **CTR1**	Hormones: ethylene; Disease resistance signling.
			C	ATN1, MRK1	Unknown.

MAPKK	Threonine/tyrosine	S/T-XXXXX-S/T	A	**MKK1**, **MKK2**, MKK6	Redox control–oxidative stress; Abiotic stresses: cold, salt, low humidity, mechanical; Biotic stresses: bacterial pathogens; Hormones: salicylic acid; Cell division.
			B	**MKK3**	Oxidative stress; Abiotic stresses: mechanical; Biotic stresses: bacterial pathogens; Hormones: jasmonic acid.

MAPKKK	Threonine/tyrosine	S/T-XXXXX-S/T	C	**MKK4**, **MKK5**	Redox control–oxidative stress; Abiotic stresses: salt; Biotic stress: bacterial pathogens; Hormones.
			D	MKK7, MKK8, **MKK9**, MKK10	Oxidative stress; Biotic stress: bacterial pathogens.

MAPK	Serine/threonine/tyrosine	TEY	A	**MPK3**, **MPK6**, MPK10	Redox control–oxidative stress; Abiotic stresses: salt, cold; Biotic stress: bacterial pathogens; Hormones: jasmonic acid.
			B	**MPK4**, MPK5, MPK11, MPK12, MPK13	Redox control–oxidative stresses; Abiotic stresses: salt, cold, low humidity, mechanical; Hormones: salicylic acid; Cell division.
			C	**MPK1**, **MPK2**, **MPK7**, MPK14	Oxidative stress; Abiotic stresses: mechanical; Biotic stresses: bacterial pathogens; Hormones: jasmonic acid, abscisic acid; Circadian-rhythm-regulated.
		TDY	D	MPK8, MPK9, MPK15/16/17/18/19/20	Oxidative stress; Abiotic stresses: mechanical; Biotic: blast fungus; Hormones: jasmonic acid.

**Table 2:** Induction of MAPKinases under metal stress. MAPK cascade modules affected by exposure to metals are categorized based upon plant species and type of kinase. Exposure to metals influences MAPKinase mRNA levels as well as the activity at the protein level.

Metal-Induced MAPKinases							

Plant	Component of MAPK cascade		Metal	Concentration	Exposure Time	Observations	Reference
*Arabidopsis thaliana*	**MAPKKK**	MEKK1	Cd	500 μM CdCl_2_	1–3 h	↑ mRNA levels	[66]
	**MAPK**	MPK3/MPK6	Cd	1 μM CdCl_2_	10 min	↑ activity	[60]
			Cu/Cd	2 μM CuSO_4_/5 μM CdSO_4_	2–24 h	↑ mRNA levels	[21]

*Brassica juncea*	**MAPK**	46 kDa MAPK	As(III)	50 μM As(III)	15 min–1 h	↑ activity	[67]

*Medicago sativa*	**MAPKK**	SIMKK	Cu	100 μM CuCl_2_	30 min	induces SAMK and SIMK	[62]
	**MAPK**	SAMK	Cu/Cd	100 μM CuCl_2_/CdCl_2_	10 min–1 h (Cu)/30 min–3 h (Cd)	↑ activity	[62]
		SIMK	Cu/Cd	100 μM CuCl_2_ /CdCl_2_	5 min–6 h (Cu)/10 min–6 h (Cd)	↑ activity	[62]
		MMK2	Cu/Cd	100 μM CuCl_2_/CdCl_2_	10 min–1 h (Cu)/10 min–3 h (Cd)	↑ activity	[62]
		MMK3	Cu/Cd	100 μM CuCl_2_/CdCl_2_	10 min–1 h (Cu)/10 min–1 h (Cd)	↑ activity	[62]

*Oryza sativa*	**MAPKK**	OsMKK4	As	50 μM As(III)	3–9 h	↑ mRNA levels	[61]
	**MAPK**	OsMSRMK2	Cu/Cd/Hg	100 μM CuSO_4/_CdCl_2/_HgClO_3_	30 min	↑ mRNA levels	[68]
		OsMSRMK3	Cu/Cd/Hg	100 μM CuSO_4/_CdCl_2/_HgClO_3_	15 min–2 h	↑ mRNA levels	[69]
		OsWJUMK	Cu/Cd/Hg	100 μM CuSO_4/_CdCl_2/_HgClO_3_	15 min–2 h	↑ mRNA levels	[69]
		OsMPK2	Cd	400 μM CdCl_2_	3–12 h	↑ mRNA levels	[70]
			Cu	100 μM CuCl_2_	3–12 h	↑ mRNA levels	[71,72]
		OsMPK3	Cu/Cd	50 μM CuCl_2_/100 μM CdCl_2_	1 h	↑ activity	[73]
			As	50 μM As(III)	30 min–9 h	↑ mRNA and activity levels	[61]
		OsMPK4	As	50 μM As(III)	30 min–9 h	↑ mRNA and activity levels	[61]
		OsMPK6	Cu/Cd	50 μM CuCl_2_/100 μM CdCl_2_	1 h	↑ activity	[73]
		40 kDa MAPK	Zn	1 mM ZnCl_2_	15 min–8 h	↑ activity	[74]
			Pb	10 mM Pb(NO_3_)_2_	30 min–8 h	↑ activity	[75]
		42 kDa MAPK	Zn	1 mM ZnCl_2_	15 min–8 h	↑ activity	[74]
			I	500 μM FeSO_4_	15–30 min	↑ activity	[76]
			Pb	10 mM Pb(NO_3_)_2_	15 min–8 h	↑ activity	[75]

*Zea mays*	**MAPK**	ZmMPK3	Cd	500 CdCl_2_	30 min–1 h	↑ mRNA levels	[77]
		ZmMPK5	Cr(VI)	250 μM Cr(VI)	30 min	↑ activity	[78]
